# Because We Love You (BeWeL): A protocol for a randomized controlled trial of two brief interventions focused on social and cultural connectedness to reduce risk for suicide and substance misuse in young Alaska Native people

**DOI:** 10.21203/rs.3.rs-3874293/v1

**Published:** 2024-01-22

**Authors:** Stacy Rasmus, Elizabeth J. D’Amico, James Allen, Cynthia Nation, Simeon John, Victor Joseph, Anthony Rodriguez, Gaby Alvarado, Allyson D. Gittens, Alina I. Palimaru, Ryan A. Brown, David P. Kennedy, Michael J. Woodward, Jennifer Parker, Keisha McDonald

**Affiliations:** University of Alaska Fairbanks; RAND Corporation; University of Alaska Fairbanks; University of Alaska Fairbanks; University of Alaska Fairbanks; University of Alaska Fairbanks; RAND Corporation; RAND Corporation; RAND Corporation; RAND Corporation; RAND Corporation; RAND Corporation; RAND Corporation; RAND Corporation; RAND Corporation

**Keywords:** Alaska Native, adolescents, suicide, alcohol, intervention, culture, social networks

## Abstract

**Background:**

Suicide among young people in Alaska Native (AN) communities was nearly unheard of through the establishment of statehood in 1959, but in the 1970s, AN suicide rates began to double every five years, with most of the increase due to suicide among 15 to 25-year-olds. From 1960–1995, the suicide rate increased by approximately 500% during this period of rapid, imposed social transition. For example, families were forced to live in settlements and children were sent to boarding schools. These disruptions increased conditions associated with suicide risk (e.g., substance use disorders, cultural disconnection), and challenged the community-level social safety net of youth protective factors that might have moderated effects of these traumas. The present study addresses the significant gap in culturally appropriate evidence-based programming to address suicide prevention among AN young people as part of aftercare. Our key research questions and methodology have been informed by AN stakeholders, and the intervention approach is Indigenous-led.

**Methods:**

Our interventions are targeted toward Alaska Native young people ages 14–24 who present with suicide attempt, ideation, or associated risk behaviors, including alcohol-related injury in the Yukon-Kuskokwim region or the Interior. In a randomized controlled trial, 14–24-year-old AN individuals will receive either BeWeL (n = 185), which comprises a 45-minute virtual cultural talk addressing family and ancestral strengths and increasing protective factors, or BeWeL plus motivational interviewing with social networks, which includes an additional 15 minutes focused on discussion of the individual’s social networks (n = 185). We will evaluate intervention effects on primary outcomes of suicide-intent risk, depression, anxiety, frequency of alcohol use, and alcohol consequences. Some of our secondary outcomes include individual and community protective factors, social networks, and awareness of connectedness.

**Discussion:**

This project has the potential to expand the range and effectiveness of suicide prevention services for AN young people and will help meet the need in Alaska to link clinical behavioral health services to AN community-based networks, and to engage local cultural resources in aftercare for individuals at risk for suicide. Findings have potential to provide practical information to advance the field of suicide prevention and enhance protective factors and resiliency among this population.

**Trial registration:**

ClinicalTrials.gov Identifier: NCT05360888

## Introduction

### Background and rationale

Alaska is a diverse and expansive state with extreme variations between ecoclimates and geographies and highly distinctive adaptations among the Indigenous peoples living since time immemorial across its 665,400 square mile landscape. Alaska Native (AN) people have historically thrived in the harshest of environmental conditions, living on the land and surviving through the millennia by drawing from deep stores of Indigenous knowledge, values, and resources that were protective against risks to life and contributed to collective well-being.

Suicide among children and adolescents in AN communities was nearly unheard of up through the establishment of statehood in 1959, but in the 1970s, AN suicide rates began to double every five years, with most of the increase due to suicide among 15-to-25-year-olds.^1^ From 1960–1995, the suicide rate increased by approximately 500%.^2^ This was a period of rapid, imposed social transition. Families were forced to live in settlements, children were sent to boarding schools, and new social, economic, and political systems^3,4^ were installed. These disruptions increased conditions associated with suicide risk (e.g., substance use disorders, disrupted social roles, and disconnection), and challenged the community-level social safety net of youth protective factors that might have moderated effects of these traumas.^5–12^ This cultural disruption in the Arctic is also associated with acculturation stress and identity struggles in young AN people^5,10,13–17^ as they dislodge age-old cultural practices, impinging on youth perceptions of Indigenous values. These imposed conditions cut young people off from traditional resilience processes^8,18–23^ with real health consequences.^16,24,25^ Indigenous young people who struggle with identity issues are more likely to use substances and exhibit suicidality.^22,26,27^ In contrast, AN young people who show communal mastery, positive family relationships, perceived community support, and feel connected to others tend to make healthier decisions about alcohol use, engage in prosocial behaviors, and have greater reasons for life; all these factors have been found to be protective against suicide.^28^

Today, Alaska has the highest state rate of suicide per capita, with rates almost twice as high as the rest of the US (27 versus 14 per 100,000 people);^29^ rates for AN people are even higher at 41.2 per 100,000,^30^ and suicide is the leading cause of death for AN young people ages 15–24.^31^ The complex issue of AN suicide among young people has yet to be adequately addressed in culturally-responsive and scientifically-grounded ways.^32^ AN individuals confront real life challenges, including traumatic experiences, unresolved grief across generations associated with forced assimilation and cultural genocide, and disruption of traditional coping resources, including intergenerational social support.^33^

There are 229 federally recognized Tribes in Alaska, and most are located in remote and rural villages accessible year-round only by small aircraft. Service delivery to address the needs of young people is compounded by remoteness and a significant lack of infrastructure and resources to address suicide risk. Twelve regional Tribal Health Organizations service these 229 Tribes and provide a range of services primarily delivered at regional hubs (i.e., the small city of Bethel) or urban centers (i.e., Fairbanks or Anchorage). Remote villages lack access to medical and behavioral health services, and residents must travel to their regional hub or an urban center to receive care from clinically trained providers. Individuals have limited inpatient options, and whether ultimately hospitalized or not, people of all ages are sent back to their home villages with limited or no connection to formal supports or follow-up to ensure continuity of care.^34^ Although most remote rural communities lack formal clinical services to address suicide risk and mental health, each Tribe and every village possess cultural strengths that have endured despite this recent history of significant disruption, providing social resources and Indigenous knowledge that can be leveraged in support of youth well-being and social connection to directly address suicide risk and promote recovery.^35^ Thus, connecting AN young people with local rural community resources immediately after a high risk behavioral event, such as a suicide attempt or an alcohol related injury, is critical. This can help increase well-being and resilience of the young person in the long-term and assist health care decision makers in understanding how to best address suicide risk in the person’s aftercare.

There is immense need in Alaska to bridge critical service gaps in Tribal health systems, and to create links between rural and urban resources that support wellness and protection from suicide risk for young people in their home communities. Additionally, there is great need for culturally-grounded interventions for AN young people.^36^ A recent meta-analysis on youth suicide prevention found that only three studies tested interventions among Indigenous people, one of which was a Yup’ik AN Indigenous-theory driven prevention model termed *Qungasvik* (phonetic: kung-az-vik) in the Yup’ik language, referencing an essential ‘toolkit’ for survival in an Arctic context.^37^ The Qungasvik prevention model, hereafter referred to by its community-advised translation as ‘Tools for Life’ (TfL), builds on existing infrastructure, expertise, and programming from each participating Tribal community, thereby involving existing culturally meaningful settings.^38^ When gaps are identified, the community-driven TfL process creates new settings and builds meaningful connections for young people to local helping and healing resources. This is critical as prior research shows limitations of interventions with Alaska Native/ American Indian (AN/AI) populations that utilize external nonlocal structures or resources that have struggled to demonstrate effectiveness, and more importantly, sustainability of the intervention over time.^39^ Growing evidence also supports ‘culture as prevention.’^40–43^ Interventions grounded in culturally-respectful and reflective structures, settings, and systems are feasible, acceptable, and effective for AN/AI populations.^44^

To date, there are no studies with AN/AI adolescents and young adults addressing suicide risk immediately after hospitalization or following high risk behavioral events that integrate culturally appropriate care with discussion of cultural strengths and social connectedness. There is a clear need in Alaska to link clinical behavioral health services meaningfully and intentionally to AN community-based structures and networks, and to engage local cultural resources in treatment and aftercare for individuals at risk for suicide. Our research team has been collaborating with AN/AI communities for over 20 years to address the significant health and mental health disparities and inequities experienced within these populations. Our work has highlighted the importance of using a community-based participatory research (CBPR) approach when developing and implementing interventions with AN/AI people, and intervening at multiple levels to address disparities.^36,44–51^ We focus on advancing strengths-based and culturally-centered approaches^38^ to reduce risk for suicide and increase protective factors,^52^ including cultural connectedness,^53^ and building reasons for life^54^ and sobriety^31^ in AN young people.

## Objectives

The present study was designed to address the significant gap in culturally appropriate evidence-based programming to address suicide prevention among AN young people as part of aftercare. Our key research questions and study methodology have been informed by AN stakeholders, and the intervention approach is Indigenous-led; we will engage stakeholders to elicit feedback in all phases to inform future improvement efforts. In this randomized controlled trial, every individual will receive culturally appropriate programming to address suicide prevention. AN young people are randomized to participate in one of two brief interventions. Both groups will receive, a brief adaptation of the evidence-based “Tools for Life” model, delivered by AN cultural leaders in a 45-minute virtual intervention utilizing modules from the Qungasvik (toolkit) manual (http://www.qungasvik.org/home/) to promote strengths, reasons for life, and social connectedness. Half of the individuals will also receive an additional 15-minute motivational intervention that addresses social networks (MISN).^55^ All participants in the project will also receive two follow up sessions via virtual visits with AN cultural leaders. The primary aim is to evaluate the comparative effectiveness of these two culturally-appropriate programs on suicide risk, alcohol use and consequences, depression, and anxiety. Our secondary aim is to determine program effects on sobriety self-efficacy, intentions to be sober, awareness of connectedness, reasons for life, reflective processes, and social network composition and structure.

## Methods

### Conceptual Model

Qungasvik ‘Tools for Life’ is based on a rural Alaska Native Indigenously-designed and delivered prevention model. Our team has written extensively about the development and delivery of a Yup’ik Indigenous theory-driven preventive intervention to reduce risk for suicide and alcohol misuse tailored specifically to the unique culture and context of rural Alaska Native communities in the Yukon-Kuskokwim subregion in southwest Alaska.^56^ What we describe here is the process we proposed to adapt the Qungasvik model into the brief BeWeL (Because We Love You) intervention.

The Qungasvik model engages three primary process steps to get to the multi-level outcomes with young people in the rural settings. The first step (Qasgiq; communal house) in the implementation of the model is to come together as cultural leaders and Elders in the community and identify key cultural teachings, practices, and activities that build strengths for young people and give them tools to survive out on the land and in their lives. The next step is identifying key strengths and resources that can be built in young people’s lives through engagement with these specific cultural teachings, practices, and activities (Protective Factors; see Table 1). This process step represents an Indigenous knowledge-driven decision to make an important paradigm shift away from the more standard practice of risk reduction approaches in suicide prevention. The Yup’ik communities and cultural leaders designing the prevention approach felt it was most important to give young people reasons to want to live and to increase their awareness of social connectedness and place in life. The third step (Module Delivery) is the community-driven practice of planning out, delivering, and reflecting upon the cultural activities and teachings that are provided to young people in a series of activities and/or meetings. Finally, the last step (Outcomes) is monitoring the effects of the community-engaged intervention implementation for young people in building strengths, increasing awareness of connectedness, and providing reasons for life and sobriety—all of which, in a Yup’ik cultural framework, ultimately contribute to collective interdependence and well-being.

### Study setting

Our study is based in two Tribal service regions of Alaska: the southwest’s Yukon-Kuskokwim (YK) and the Interior. The YK region has the state’s highest rural Alaska Native population with over 26,000 tribal members residing in 56 villages across a geographic service region that is the size of the state of Nebraska. The Yukon-Kuskokwim Health Corporation (YKHC) is a single-payer health system serving the AN communities in the YK region and is located in the regional hub of Bethel, Alaska (pop. 6600). However, there is no road system connecting any of the communities or the regional hub to outside services in the major cities. Mental health services are scarce outside of the regional hub. Young people in crisis are most often flown into Bethel to be seen in the emergency department by 24-hour on call behavioral health staff, and those in need of ongoing support and care have access primarily through videoconferencing technology with mental health staff in Bethel.

Our second study setting is the Interior region that is serviced by the Tanana Chiefs Conference (TCC), located in Fairbanks, AK. TCC provides a single-payer health system and social services for its 16,000 members throughout the 6 sub-regions and 42 tribes of Interior Alaska. The TCC service region covers an area of 235,000 square miles in interior Alaska, which is equal to about 37 percent of the entire state, and just slightly smaller than the state of Texas. Services for rural residing young people in the Interior are similar in terms of gaps and needs as described above for the YK region. Young people in crisis or engaging in risk behaviors, such as heavy drinking, in Fairbanks and in the rural communities are often sent to the emergency department at the Fairbanks Memorial Hospital. From there, they are either referred out to one of only two psychiatric level inpatient facilities, both located in Anchorage, or they are discharged with a referral back to TCC. Those living in the rural communities have very limited access to follow-up and aftercare. Our study will recruit in Fairbanks, Alaska with an initial focus on engaging emergency department staff at the local hospital along with outpatient behavioral health and social work staff at the local Tribal health and social service organizations. Additionally, we will recruit from other youth-serving agencies including child welfare, the youth shelters, and the AN-serving charter and boarding schools within the region. We will continue to broaden recruitment out to the rural communities to meet enrollment goals.

### Participants

Our interventions are targeted toward Alaska Native young people ages 14–24 (*N* = 370) who present with suicide attempt, ideation, or associated risk behaviors, including alcohol-related injury in the YK region or the Interior. We focus on this age group because suicide is the leading cause of death.^31^

### Recruitment and consent/assent

In the YK, our study will recruit in Bethel, Alaska with an initial focus on engaging clinical staff and providers in key positions within the Tribal health organization who come into contact with AN young people who are at risk, such as the emergency on-call behavioral health clinicians and hospital social workers. We will also recruit from other youth-serving agencies and organizations in Bethel including child welfare, youth residential facilities, and schools. We will broaden recruitment efforts to rural communities as needed to meet enrollment goals. In the Interior region, our study will recruit in Fairbanks, Alaska with an initial focus on engaging emergency department staff at the local hospital along with outpatient behavioral health and social work staff at the local Tribal health and social service organizations. Additionally, we will recruit from other youth-serving agencies including child welfare, youth shelters, and the AN-serving charter and boarding schools within the region. We will continue to broaden recruitment out to rural communities to meet enrollment goals.

When a young person indicates interest and gives permission, the provider will complete an online consent to contact form. This form collects contact information so that a staff member can contact the parent and/or young person to discuss the project further, obtain consent, and address any questions. Once consent is obtained from those who are 18 or older or from the parent (for those 17 and younger), and we receive assent from those 17 and younger, the young person will then receive a link to complete the baseline survey.

### Interventions

In our prior long-term CBPR research experience implementing randomized controlled trials (RCTs) in these communities, our Tribal partners have found our study designs and treatments ethical, particularly for interventions with the potential to reduce risk for suicide in young people. We have a strong evidence base supporting the positive effects of the Qungasvik/TfL approach and for the discussion of social networks among this population.^38,57–61^ In this RCT, all young people receive BeWeL (Because We Love You) as described below, and half will be randomized to also receive the motivational interviewing social network intervention (MISN). All young people in the study also receive two follow up virtual visits. Tables 2 and 3 summarize the BeWeL brief intervention activities and protective factors delivered in each section of the 45-minute cultural talk.” Sections are patterned according to four different times during daylight, itself a salient orienting feature that can vary in these communities by over 20 hours over the year. Table 4 summarizes the MISN brief intervention activities.

#### Brief Adaptation of Qungasvik into BeWeL and Cultural Adaptation to a Different Alaska Native Cultural-Linguistic Group

We proposed to engage AN cultural leaders in the brief intervention adaptation process to accomplish two levels of cultural adaptation to the established Qungasvik model. We initially engaged Yup’ik Alaska Native cultural leaders and young people to adapt the measures and the delivery modality to be more closely tailored to young people who are coming into the intervention from clinical settings. We collectively evaluated the Qungasvik Protective Factors (Table 1) and the Qungasvik Teachings, to co-produce a brief intervention implementation process model delivered virtually by trained AN cultural leaders. Table 2 provides the summary of the BeWeL intervention activities alongside the adapted Qungasvik module.

**Figure 1:**
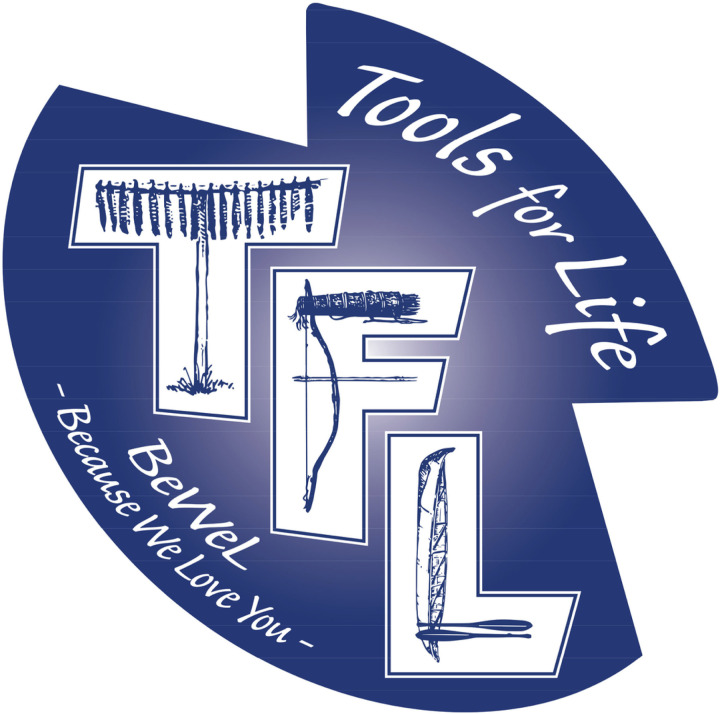
Legend not included with this version

**Figure 2:**
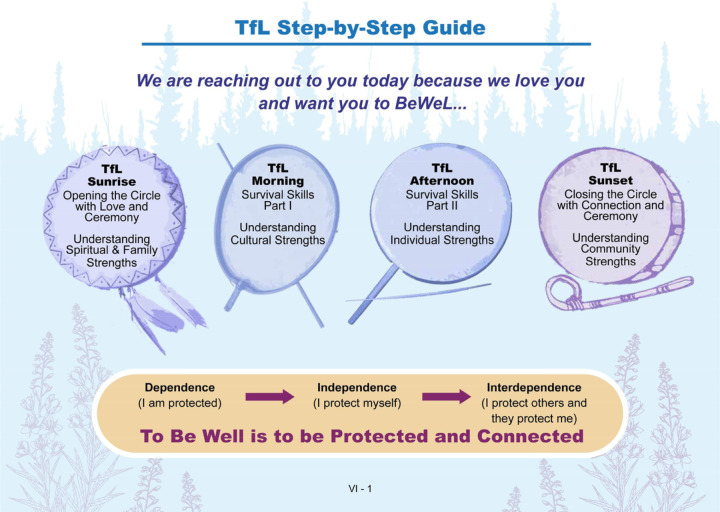
Legend not included with this version

The brief adaptation focuses on delivery of protection and tools for life via a four-part virtual ‘cultural talk’ that draws from teachings based on ancestral strengths, kinship, subsistence practices, survival skills, and social connections with Elders. Protective factors are built through stories, teachings, and reflections with young people by AN community and cultural leaders during these cultural talks. Early on in the brief intervention adaptation process, it was suggested that we rename the brief version of TfL to start with a culturally meaningful phrase, “Because We Love You” (shortened to BeWeL). Elders will often use this phrase when speaking with young people; “We share these words because we love you.” BeWeL as a brief version of TfL is story-based in its delivery and continues to build protective factors that provide tools for life and awareness of connectedness for young people. Figure 1 is the BeWeL logo designed by Garry Utermohle with extensive feedback from young people and the community. Figure 1. **BeWeL project logo (designed by Garry Utermohle)** The cultural teachings are tailored to the community and region where the young person lives. Protection is considered on multiple levels, including the family, community, and spirituality of a young person along with their own inherent strengths as individuals. Ceremony and prayer are integrated into the virtual space to deepen the sacredness of the work being done and the connections established through space and in communal spirit. Figure 2. **Tools for Life step-by-step process** Fig. 2 represents the co-produced Brief TfL step-by-step process. The drums each represent one stage of change to explore and reflect upon with a young person during the 45-minute virtual cultural talks. The drums and cultural talk are organized based on an Indigenous conceptualization of the stages of change that begins with establishing a spiritual connection, through prayer and/or ceremony (smudging). A young person is then engaged in reflection on family and ancestral strengths to build awareness of connectedness to the historical resiliencies that they inherit from generations past and present. The cultural talk process then asks young people to consider tools in their toolkit for surviving out on the land. Nature is presented as a primary healing resource and a provider of sustenance for the mind, body, and spirit. Connecting to the land and animals extends a young person’s relational universe. Next, the skills needed to navigate out on the ice and maintain respectful relationships with the animals are translated into everyday life. This translation emphasizes how these skills can also guide a young person through challenges and dangers in their personal lives and can ground their relationships with peers and family. The cultural talk process concludes with an acknowledgement of the central role and purpose that each young person has as part of their own home community, and within the context of the larger Alaska Native community, where they are loved and valued. The stages of change follow the same directional flow at the core of the Qungasvik intervention efforts in developing reasons for life. Young people move through a process of first understanding their dependence on the connections and strengths of their kinship networks. They then move towards independence through an awareness of how protective their culture is and how powerful they are in living their cultural ways of life, and ultimately arrive at an understanding of their interdependence, appreciating both their interconnection and their own inherent, community acknowledged value as a young person in an AN context.

In parallel process of cultural adaptation, our team engaged AN cultural leaders from the Interior of Alaska who belong to Tribes representing Koyukon Athabascan and Gwich’in cultures to assess the acceptability and cultural appropriateness of the BeWeL model for young people from communities culturally distinct from Yup’ik Alaska Native settings, and to provide recommendations for cultural adaptation in content and process of the intervention. The BeWeL model is a flexible, adaptable approach that emphasizes the function of intervention activities in their protective factors delivery over the specific form the activity takes. The protective factors are culturally rooted in Yup’ik communities, but the roots run to the core of AN Indigenous values and are broadly interconnective across different AN cultures and contexts. The teachings can be adapted in form to fit other AN cultural contexts. The cultural leaders conducting the cultural talks in the YK and the Interior bring their backgrounds and connections from their Yup’ik or Athabascan traditions, matching the intervention form of content and protocol to the needs and orientation of each young person, while maintaining the focus of each section. Through these efforts, BeWeL has a cultural congruence across each of the study settings in Alaska.

### BeWeL plus MISN

Our prior research has shown the importance of leveraging healthy social networks and cultural connectedness among AN/AI young people to help decrease suicide risk and alcohol and cannabis use.^59,62^ For example, our data show that urban AN/AI emerging adults with higher proportions of network members engaging in traditional practices and who do not report heavy alcohol use, regular cannabis use, or other drug use are less likely to report intentions to use cannabis or drink alcohol in the future.^59^ Similarly, our work has highlighted that supportive social networks increase protective factors from suicide and alcohol use among rural Yup’ik young people.^62^ Based on evidence suggesting the important role that social networks play in changing young people behaviors, particularly in terms of promoting protective factors and reducing risk factors for substance misuse, we proposed a comparative effectiveness RCT study design where half of the young people who enroll in BeWeL will be randomly assigned to receive an additional 15-minute MISN intervention with the cultural leaders following their cultural talk.

**Figure 3:**
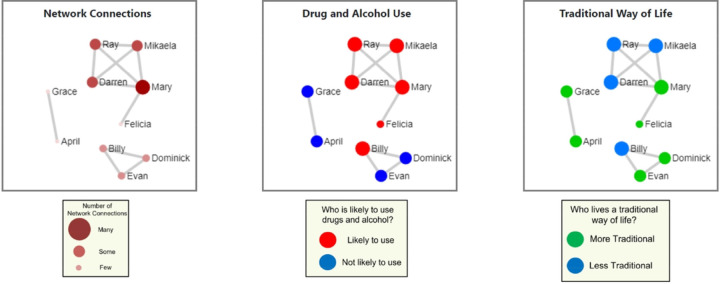
Legend not included with this version

The MISN brief intervention is specifically focused on helping young people think through how to decrease risk and increase support in their networks to help them make healthy choices. Leveraging healthy social networks and cultural connectedness among AN/AI young people can help decrease suicide risk and alcohol use, and AN/AI young adults additionally describe the social network (SN) visualizations used in the intervention as engaging and helpful.^63^Fig. 3. **Example of a social network visualization** Fig. 3 provides a visualization example. In the visualization presented to the young person, network members are represented by circles (nodes), and lines between nodes represent network contacts who interacted with each other in the past two weeks. The “Your Network” visualization shows names of people the participant reported interacting with in the past two weeks. The centrality of nodes is conveyed by calibrating node size and color with degree centrality (number of connections the young person had in the past two weeks for a particular node), and line thickness with the participant’s rating of relationship strength between the two nodes. Participants often label groups of members (e.g., the party friends). “Drug and Alcohol Use” shows larger red nodes for people who the young person rates as likely to use substances in the next two weeks and smaller blue nodes for those who are unlikely. “Traditional Way of Life” shows larger green nodes for people who engage in traditional practices and live a more traditional way of life, and smaller blue nodes for people who do not. The accompanying discussion to viewing the visualizations, as guided by the cultural leader, will focus on personal choices, and will draw on roles that networks play in making healthy choices, such as staying connected culturally and ways to increase resilience. Participants will discuss how networks affect choices, and how to address negative influences while retaining and increasing positive elements of their networks. Networks will be discussed again in the two virtual follow up sessions to address where the individual can get support in making healthy choices and engage in traditional practices in their communities.

### Intervention fidelity

Fidelity to a culturally-based intervention includes adherence to culture-specific practices that include guidance from Elders and the cultural protocols of engagement rooted in traditional organizational practices. Fidelity to motivational interviewing and the protocol will be monitored through checklists completed by facilitators and through observation of some of the virtual sessions. We will measure adherence to the BeWeL and MISN brief intervention protocols with fidelity checklists with response options ranging from “completely covered” to “not at all covered”.^64–66^ Furthermore, before facilitators go into the field, we will provide extensive training on motivational interviewing and the BeWeL and MISN interventions. By the end of training, facilitators will follow the protocol and have a high rate of MI-consistent behaviors and adherence to the protocol. Throughout the study, we will provide supervision and feedback to facilitators on protocol adherence and offer booster training whenever adherence to fewer than 80% of checklist items is observed.

### Trial design

This is an RCT with block randomization of 370 young people to BeWeL or BeWeL + MISN. As noted, every young person will receive a cultural intervention, and half will also receive a brief discussion about their social networks using the social network visualization.

### Randomization process and study flow

**Figure 4:**
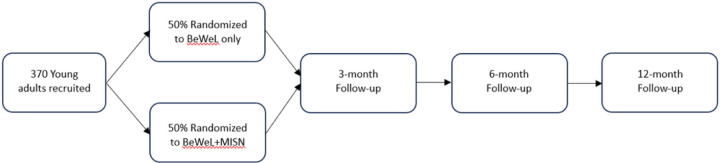
Legend not included with this version

**Figure 5:**
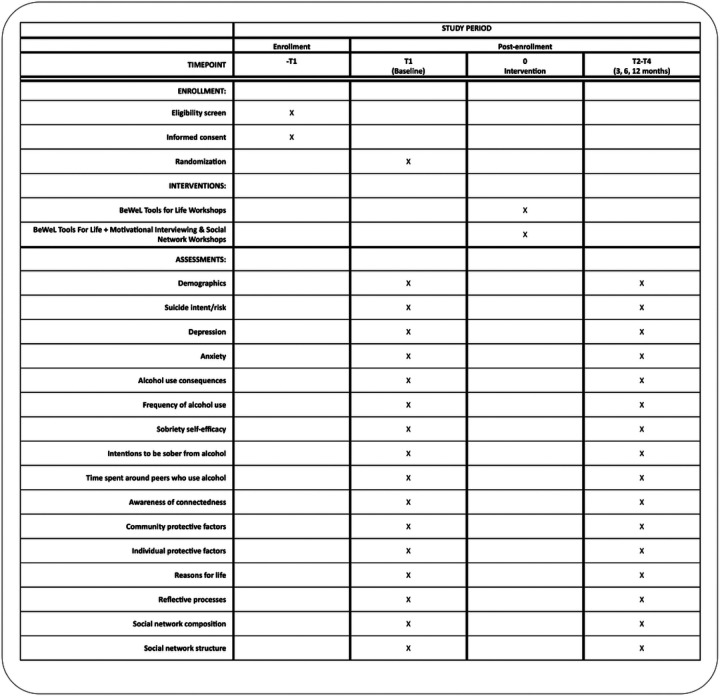
Legend not included with this version

Once consent is obtained, the young person will receive a link to complete the baseline survey. Upon completion of the baseline survey, each participant will be randomized to either BeWeL or BeWeL + MISN. A BeWeL staff member will be notified and provided the randomized treatment group allocation for the young person. The BeWeL staff member will contact the participant to provide the BeWeL or BeWeL + MISN intervention. All participants will be asked to participate in two virtual follow up visits at 2 weeks and 6 weeks after both conditions. They will also be invited to complete 3-, 6-, and 12-month follow up surveys. For each survey administration, detailed information will be obtained on how to reach the respondent (primary address, email, home phone, cell phone, parents’ phones, etc.). Figure 4. **Participant flow through the study** Fig. 4 depicts participant flow through the study, Fig. 5. **SPIRIT diagram** and Fig. 5 contains a SPIRIT (Standard Protocol Items: Recommendations for Interventional Trials) flow diagram of the RCT schedule of enrollment, interventions, and assessments.

### Measures

Measures were selected based on prior use, acceptability, and strong psychometric properties in AN/AI or other Indigenous communities. Outcomes will be assessed at baseline, and at 3-, 6-, and 12-months.

### Primary outcomes

#### Suicide intent/risk.

The Suicidal Ideation Attributes Scale (SIDAS)^67^ is designed to screen individuals in the community for presence of suicidal thoughts and assess the severity of these thoughts. It consists of five items, each targeting an attribute of suicidal thoughts: frequency, controllability, closeness to attempt, level of distress associated with the thoughts, and impact on daily functioning. Responses are measured on a 10-point scale. Items are coded so that a higher total score reflects more severe suicidal thoughts.

#### Depression.

Depression in the last two weeks is assessed using a sum from the 9-item Patient Health Questionnaire (PHQ-9)^68^ (0=”not at all” to 4=”nearly every day”; α = 0.92).

#### Anxiety.

Anxiety in the last two weeks is assessed with the 7-item Generalized Anxiety Disorder scale (GAD-7)^69^ (0=”not at all” to 3=”nearly every day”; e.g., feeling nervous, anxious, or on edge; α = 0.95).

#### Alcohol consequences.

Consequences from alcohol in the past three months (e.g., passed out) will be assessed by summing 4 items (1= “never” to 7= “20 or more times”) utilized in previous work with this age group.^70^

#### Alcohol use.

We will assess alcohol use at each assessment with Monitoring the Future (MTF) items.^71^ The consistency and reliability of these measures have been shown in numerous studies.^72–74^ At baseline, we will measure lifetime (0 = 0 times, 1 = 1 or 2 times, 2 = 3–9 times, 3 = 10–19 times, 4 = 20–39 times, 5 = 40–99 times, 6 = 100 + times), 3-month (0 = none, 1 = 1 time, 2 = 2 times, 3 = 3–5 times, 4 = 6–9 times, 5 = 10–19 times, 6 = 20–30 times, 7 = 31 + times) and 30-day use (number of days). At follow-up time points, we will measure past 3-month and past 30-day use.

### Secondary outcomes

#### Sobriety self-efficacy.

Participants report the likelihood that they could stay sober: 1) in their community; 2) if they are around friends who are drinking; and 3) if their best friend is drinking. Higher scores indicate higher self-efficacy.^75^

#### Intentions to be sober.

Participants will be asked if they think they will be sober from alcohol in the next month.

#### Time spent around peers who use alcohol.

Participants will be asked how often they are around peers who drink alcohol from “Never”=0, “Hardly ever”=1, “Sometimes” =2, “Often”=3.^76^

#### Awareness of connectedness (ACS).

Nine items focus on assessing awareness of self as a member of a broader human and natural community, including an awareness of connections between one’s own well-being and the well-being of other entities in the various ecological spheres that one occupies (e.g., When I do good things for my community good things happen to me). The ACS assesses the degree to which a person endorses the concept of interrelatedness between self, family, community, and natural environment.^53^ Participants respond with a slider from 0 (not at all) to 20 (a lot).

#### Community and individual protective factors.

^62^ These items focus on protective factors that occur in the community (e.g., people supported and helped me if I needed it), family, (e.g., my family teaches good values), and within the individual (e.g., working together with friends I can solve many of my problems). Participants respond with a slider from 0 (not at all) to 20 (a lot).

#### Reasons for life.

Reasons for Life (RFL) comprises three subscales: *Cultural and Spiritual Beliefs, Efficacy Over Life Problems*, and *Others’ Assessment* (e.g., My Elders teach me that my life is valuable). Higher scores on the RFL are hypothesized to indicate more positive attitudes toward life and higher levels of protection from suicide.^77^ Participants respond with a slider from 0 (not at all) to 20 (a lot).

#### Reflective processes.

^78^ The Reflective Processes scale taps a culturally patterned type of awareness (ellangneq) used in thinking over potential negative consequences of alcohol misuse engaged by Alaska youth when considering reasons not to drink with eight items (e.g., My friends and I talk about how we have better things to do than go drink). Participants respond with a slider from 0 (not at all) to 20 (a lot).

#### Social Networks.

##### Social Network Composition and Structure.

Participants will complete network interviews at baseline and all follow-up assessments to measure network characteristics and changes using procedures from our previous work^79,80^ and standard procedures for collecting and analyzing personal networks.^81–83^ Participants will be asked to name up to 10 network contacts (“alters”).^84,85^ Participants will answer questions about each alter (e.g., demographics, relationship quality, likelihood to use substances) to produce raw data for network composition measures (e.g., percent who engage in substance use).^86^ Participants will identify ties among alters to produce raw relationship data to measure network structure (density of ties, average centrality of network members who use substances, etc.).^87,88^

### Sample size and power

We conservatively compute estimated power for the primary study aim to assess the comparative effectiveness of the BeWeL vs. BeWeL + MISN intervention based on the final projected sample size accounting for attrition at the 12-month follow-up. Based on our previous work,^89,90^ we estimate 80% retention at the 12-month follow-up, which will be a final sample of 296 participants at the end of the intervention (n = 148 per arm of the intervention). With these sample sizes, assuming a correlation between repeated assessments of 0.50, four timepoints, and alpha of .05, we have 80% power to detect a standardized effect size (d) of 0.31 between groups and standardized effects size (*d*) of .22 within groups; thus, we are powered to detect small effects using conventional standards for Cohen’s *d*.

### Data collection methods

Data at all time points (baseline, 3-, 6-, and 12-month) will be collected using web-based surveys. All Record Management System functions will be conducted on RAND’s Survey Research Group’s secure network segment. Computers on the secure network segment are isolated from the rest of the RAND network (i.e., no Internet access, e-mail or file sharing between these computers and the unclassified network) minimizing the possibility of infection by malicious software and unintentional exposure of sensitive data. The computers on the segment will also employ standard password protection along with file and folder permissions limiting access to appropriate project staff.

### Aims

We plan to compare the effectiveness of BeWeL versus BeWeL + MISN over a one-year period. In our primary aim, we will compare outcomes between the two groups at 3, 6, and 12 months on suicide risk, alcohol use and consequences, depression, and anxiety. In our secondary aims, we will evaluate the comparative effectiveness of these two groups on sobriety self-efficacy, intentions to be sober, awareness of connectedness, reasons for life, reflective processes, and support from social networks. Finally, we will use qualitative data to provide an in-depth understanding of patient satisfaction of the intervention and participants’ perspectives of culturally centered programming, identifying components valued by the participants and associated with their outcomes.

### Statistical methods

We will conduct descriptive statistics and examine missing data. Frequencies will be examined for evidence of sparseness for categorical data and for non-normality (using plots, examination of skewness, kurtosis, etc.) for continuous variables. Where sparseness exists in categorical variables, we will collapse as necessary to produce cell sizes sufficient for analysis. Where non-normality is evident, variables may be transformed. Outliers may be recoded or omitted if necessary. Missing data will be dealt with using multiple imputation and/or full information maximum likelihood estimation. The N of 370 was determined in a priori power analyses to be sufficient to detect small to moderate intervention effect sizes for all primary and secondary outcomes.

### Baseline equivalence across experimental groups

We will evaluate comparability of experimental groups with respect to potential confounders. Categorical methods of analysis (e.g., cross tabulations, chi-square) will be used to compare groups for discrete data (e.g., employment, school status). ANOVA or t-tests will be used to test for homogeneity of groups for continuous data at baseline. If a statistically significant difference is found, the covariates will be included in all subsequent analyses. If we observe considerable differences in the experimental groups that cannot be adequately accounted for with the addition of model covariates, we will develop analytic weights using propensity methods to balance the groups.

### Primary and secondary outcomes

As a first step, we will examine descriptive statistics. Frequencies will be examined for evidence of sparseness for categorical data and for non-normality (using plots, examination of skewness, kurtosis, etc.) for continuous variables. Where sparseness exists in categorical variables, we will collapse as necessary to produce cell sizes sufficient for analysis. Where non-normality is evident, variables may be transformed or handled through appropriate model estimation. Outliers may be recoded or omitted if necessary. Results from our examination of baseline equivalency will inform the inclusion of covariates in all subsequent analyses in addition to standard covariates. To examine longitudinal change and comparisons between BeWeL and BeWeL + MISN on outcomes, we may use more than one method to analyze the data. One option is to use SAS Proc Glimmix, which can handle both continuous and categorical outcomes as well as accounting for overdispersion and/or zero-inflation as needed using restricted maximum likelihood estimation. Alternatively, we will work within a multigroup latent growth model framework to examine change over the 12-month period using maximum likelihood estimation for continuous outcomes or weighted least square mean and variance (WLSMV) for categorical outcomes as implemented in Mplus. Depending on the preponderance of zeros, type of outcome, and distribution of outcomes, we have the flexibility of using alternative models (e.g., Poisson, zero-inflated Poisson, two-part semicontinuous). In addition to modeling change over the entire study period, we will examine outcomes at each time point (3, 6, and 12 months) using traditional analytic methods (e.g., regression, t-tests). Analyses will be by intention to treat; we will attempt to follow up with all individuals, regardless of attendance. We will examine overall attendance of BeWeL and BeWeL + MISN, and model attendance to detect factors that alter the probability of attending using ordinal logistic regression approaches.

### Qualitative methods

Forty participants (20 from each intervention group) will be randomly selected to complete qualitative interviews at 3-month follow up to understand patient satisfaction with the intervention. All interviews will be audio-recorded and transcribed. Verbatim transcripts will be uploaded to NVivo,^91^ a collaborative qualitative analysis software. At least two coders will code transcripts using both inductive and deductive coding.^92,93^ Open and in vivo coding will be used to establish categories and themes.^94,95^ Open coding refers to labeling interview content based on dimensions emerging from it.^93^ In vivo coding means assigning code labels using words or short phrases directly from the text.^93^ Coding will first occur on a small random sample (20%) of transcripts. Discrepancies will be resolved through team reconciliation. Once a final code list is agreed, we will proceed coding the rest of the transcripts, until we reach reliability (Cohen’s kappa) of at least 0.70.^96,97^

### Limitations and alternative methods considered

There are some important limitations to our work. First, it is difficult to recruit young people in crisis for multiple reasons. For example, parents may be scared and/or refuse services, and young people cannot be reached while in an inpatient setting. Thus, we have a plan in place to also recruit more broadly across other settings, including hospitals and clinics, outpatient mental health facilities, foster care, schools, and other community settings. Second, BeWeL is a virtual adaptation of a community-based intervention that engaged young people in person in cultural activities. However, young people, especially now following COVID-19 shutdowns, are not engaging in community settings as frequently and many stay at home during community events and engage primarily in online spaces. Thus, BeWel provides a unique opportunity to reach many young people despite not engaging in cultural activities in person. We will also have to think carefully about retention as young people may move around, including out of state, and often change their cell phone numbers. However, our survey research group has an excellent track record of obtaining high retention with difficult to reach populations, and we have several procedures in place (e.g., obtaining contact information at every follow up, and getting additional numbers for contacts) that will help increase retention. Finally, we are not using a neutral control group, thus conclusions about efficacy of the intervention are in reference to our active control group. However, our work in these communities over the last two decades and discussions with our Tribal approval boards emphasized the importance of ensuring that all participants in the RCT received some type of cultural programming.

## Discussion

This is the first study to test the effectiveness of a culturally-grounded and Indigenous theory-driven intervention with a social network intervention intended to build multi-level strengths and protective factors across culturally distinct contexts for AN young people from two regions of Alaska. The BeWeL intervention draws from teachings based on ancestral strengths, kinship, subsistence practices, survival skills and social connections with Elders, and is the first virtual intervention to address suicide risk among AN young people in the state of Alaska. The MISN component specifically focuses on increasing protective social networks. The current study will be able to address important questions about the protective role of social networks for AN young people, providing an understanding of the amount of support they receive to engage in traditional practices and make healthy choices around substance use. Furthermore, this study will address critical gaps in continuity of care in Alaska, specifically focusing on rural residing AN young people who lack access to mental health professionals and clinical services and rely on cultural and community resources.

**Figure 6:**
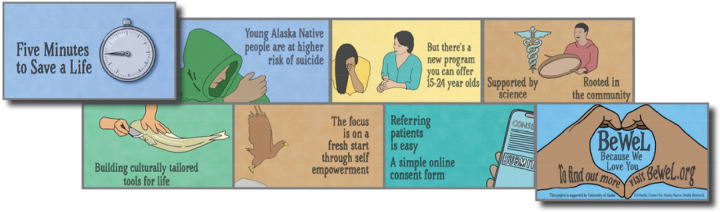
Legend not included with this version

This clinical trial will compare the effectiveness of BeWeL and BeWeL plus MISN by examining outcomes over a 12-month period. We will assess not only effects of these two culturally appropriate interventions on subsequent suicide risk and alcohol use, but we will also examine whether protective factors such as awareness of connectedness and support from social networks are affected by the interventions. Overall, this study will deepen young people’s understanding of who they are, where they come from, and will help them develop healthy connections to their ancestral past. Young AN people are in tremendous need of opportunities to learn more about their culture and ancestral ways of living as traditional ways of living were disrupted through colonial processes. Traditionally, the relationships that young people formed out on the land, living a subsistence way of life through hunting, fishing, and gathering were central to cultural identify formation and holistic well-being on multiple levels. BeWeL seeks to meet young people where they currently are at culturally, in a virtual space, to share cultural teachings and make intergenerational connections that can bridge the gap between the differing worlds that AN young people inhabit today. Figure 6. **Stills from the BeWeL recruitment video, Lifeline** (https://www.bewel.org/referrals) Fig. 6 highlights stills from an award-winning video created by the team and community to educate providers about the project and how to refer young people to our program.

Our previous work has highlighted the importance of culture in increasing resilience and protective factors among AN/AI young people,^11,38,56,59,89^ and the current study will advance the paradigm shift in suicide prevention^98^ to focus on community-level protective factors and cultural strengths and reasons for life. There are several innovative aspects to this study, including virtual implementation of this cultural intervention to address suicide risk and alcohol use, a focus on creating intergenerational connections, and leveraging of healthy social networks. One of the most novel aspects of this study is its engagement of a positive Indigenous psychology approach, developed and driven by AN cultural leaders and community stakeholders. These leaders conceptualized stages of change for suicide prevention in AN young people that focuses on developing awareness of connectedness, interdependence, and a feeling of being loved and valued by their community and of having an important role in their community giving them purpose in life.

There is a tremendous contemporary Indigenous community interest in the affirming potential of ‘culture as prevention.’ The current study addresses a critical programming need for culturally-based suicide prevention strategies for AN young people by using Alaska Native culture as its central organizing principle. In doing this, it emphasizes the importance of community-level protective factors and increased supportive social connections based in and understood through an Alaska Native cultural framework as a solution to address suicide among young people.

## Figures and Tables

**Table 1:** Protective Factors

Protective Factors Delivered	Definition	Yup’ik Term
Affection/Recognition	Recognize and give praise for good behavior and efforts toward helping the family.	*Quyavikluku*
Awareness	Being aware of the consequences of one’s own actions and how they affect family and community.	*Ellangaq*
Being Treated as Special	Encourage youth to find and fulfill their path by guiding them in a specific direction or cultural role.	*Pirpakumalria*
Clear Limits and Expectations	Define acceptable behavior for youth. Expectations are consistently repeated and enforced.	*Alerquutet*
Communal Mastery	Confidence that personal problems can be solved by working together with other people, such as family and community members.	*Kayuukut*
Family Role Models	Family members lead by example and encourage others to be sober.	*Takarnarqellria*
Giving	Sharing with others and contributing to family and community. This cultivates a sense of purpose and responsibility.	*Naklegtalria*
Role Model	Non-family members such as elders and community leaders who work hard, live a good, clean and sober life, and share what they know with others.	*Nukalpiaq*
Opportunities	Positive things for youth to do to prevent boredom and increase a sense of belonging and purpose.	*Ciunerkaat ikirrluku*
Safe Place	Places that are free from substance abuse and violence. Abusive behavior is not tolerated.	*Qinuilnguq*
Self-Efficacy	A person’s belief and confidence that he/she can solve their own problems.	*Piyugngaunga*
Village Rules	Enforcement of local alcohol laws and youth curfew laws.	*Nunam Inerquutai*
Wanting to be a Role Model	Setting an example for others by choosing to live life in a good way.	*Ciuliqagcugtua*

**Table 2.** BeWeL Brief Intervention Activities

Section	Summary of Activities	Adapted Qungasvik Modules
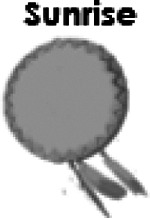	**Opening the Circle** Understanding spiritual and family strengths Open·with prayer, song, smudge Establishing a connection Kinship terms Sharing stories of strong ancestors	Kinship Terms Stories of Strength Where We All Come From
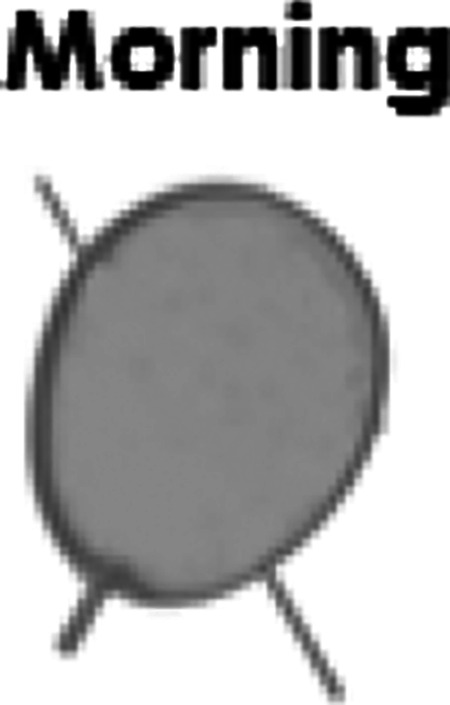	**Survival Skills (Part 1)** Understanding cultural strengths Tools for protecting yourself on in the day to day How to identify, avoid and get out of dangerous situations Sharing stories of surviving dangerous situations	Survive Skill s to Build Confidence The Land Provides for Us Building Tools for Life The Whole Human Being
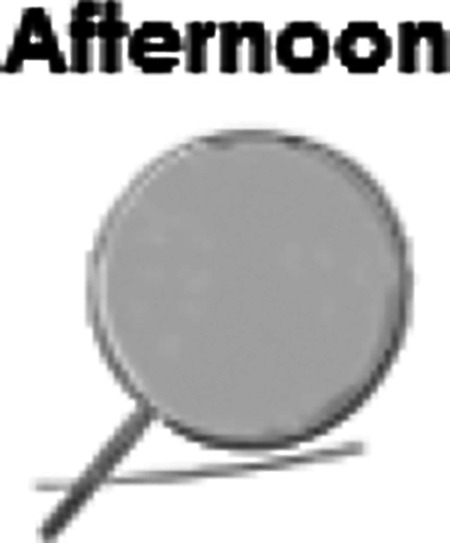	**Survival Skills (Part 2)** Understanding individual strengths Tools for protecting yourself in life and relationships Sharing stories about relationship struggles or loss	Surviving Your Feelings Be a Friend Strong and Sober Relationships fora Good Life The Food We Eat
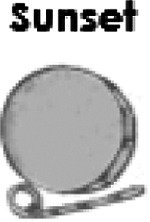	**Closing the Circle** Understanding community strengths Getting plugged in to your community Community safety net Close with ceremony: prayer, song, or good words	How Powerful You Are Prayer Walk Crisis Response Team Preparing for the Journey – this is a beginning!

**Table 3.** Protective Factors Delivered during the BeWeL Intervention

Protective Factors Delivered	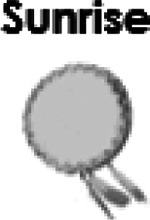	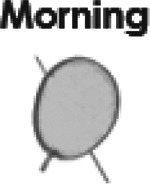	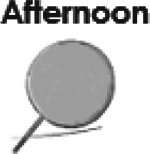	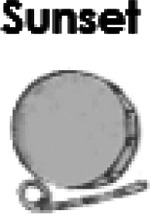
Affection/Recognition		✓	✓	
Awareness	✓	✓		✓
Being Treated as Special	✓			✓
Clear Limits and Expectations			✓	
Communal Mastery				✓
Family Role Models	✓			
Giving			✓	
Healthy Relationships to the Past	✓			
Opportunities		✓		✓
Safe Place		✓		✓
Self-Efficacy	✓	✓		
Village Rules			✓	
Wanting to be a Role Model			✓	✓

**Table 4:** Brief Motivational Interviewing and Social Network Activities

Summary of Activities	Example questions
**Discuss social network and connections people have with one another**	***What do you notice about the connections people have with each other?*** ***Are there important people in your life missing from the diagram?***
Discuss people in network that may use substances and how those people may influence the participant	*What do you notice about how people who may use substances are connected to each other?* *How do the people in your network who use substances influence you?*
Discuss how participant can avoid using substances if they don’t want to	*If you wanted to avoid drugs or alcohol, what would you have to change in your networks?* *Who would be supportive of you making this change?*
Discuss people in network who live a more traditional way of life and how those people can influence participant	*What do you notice about the people in your network who choose to live a cultural or traditional way of life?* *What is different about them?*
Discuss how participant can get support to live a more traditional life and connect with their community	*If you wanted to live a cultural or traditional way of life more than you do now, how would you make this happen?* *Who in your social network or community would support this?* *How can you connect more with your community to help you if you want to live a more cultural or traditional way of life?*

## Data Availability

The datasets generated and analyzed during the current study are not publicly available pursuant to Title 45 CFR 46 which recognizes that American Indian and Alaska Native Tribes have the authority to make the decisions and set policy in the protection of human subjects involved in research taking place with members of federally-recognized Tribes. Datasets may be made available from the corresponding author on reasonable request in the most user-friendly, accessible, secure, and ethical format. We will take necessary steps to ensure we adhere to the PCORI guidelines on sharing of data, in collaboration with our tribal partners.

## Abbreviations

ACS: Awareness of connectedness
AN/AI: Alaska Native/American Indian
AN: Alaska Native
ANOVA: Analysis of variance
CBPR: Community-Based Participatory Research
GAD: Generalized Anxiety Disorder
MI: Motivational Interviewing
MISN: Motivational Interviewing Social Network
MTF: Monitoring the Future
PHQ: Patient Health Questionnaire
RCT: Randomized Controlled Trial
RFL: Reasons for Life
SIDAS: Suicidal Ideation Attributes Scale
SN: Social Network
SPIRIT: Standard Protocol Items:Recommendations for Interventional Trials
TCC: Tanana Chiefs Conference
TfL: Tools for Life
WLSMV: Weighted Least Square Mean and Variance
UAF: University of Alaska Fairbanks
YK: Yukon-Kuskokwim
YKHC: Yukon-Kuskokwim Health Corporation
YKHC HSC: Yukon-Kuskokwim Health Corporation Human Studies Committee
